# Bioactive Steroids from the Formosan Soft Coral *Umbellulifera petasites*

**DOI:** 10.3390/md14100180

**Published:** 2016-10-11

**Authors:** Chiung-Yao Huang, Che-Wei Chang, Yen-Ju Tseng, Jessica Lee, Ping-Jyun Sung, Jui-Hsin Su, Tsong-Long Hwang, Chang-Feng Dai, Hui-Chun Wang, Jyh-Horng Sheu

**Affiliations:** 1 Department of Marine Biotechnology and Resources, National Sun Yat-Sen University, Kaohsiung 804, Taiwan; huangcy@mail.nsysu.edu.tw (C.-Y.H.); m005020026@student.nsysu.edu.tw (C.-W.C.); pit0424@yahoo.com.tw (Y.-J.T.); pjsung@nmmba.gov.tw (P.-J.S.); x2219@nmmba.gov.tw (J.-H.S.); wanghc@kmu.edu.tw (H.-C.W.); 2 Department of Medicinal and Applied Chemistry, Kaohsiung Medical University, Kaohsiung 807, Taiwan; jessicalee960410@gmail.com; 3 National Museum of Marine Biology & Aquarium, Pingtung 944, Taiwan; 4 Graduate Institute of Natural Products, College of Medicine, Chang Gung University, Taoyuan 333, Taiwan; htl@mail.cgu.edu.tw; 5 Research Center for Industry of Human Ecology and Graduate Institute of Health Industry Technology, Chang Gung University of Science and Technology, Taoyuan 333, Taiwan; 6 Department of Anesthesiology, Chang Gung Memorial Hospital, Taoyuan 333, Taiwan; 7 Institute of Oceanography, National Taiwan University, Taipei 112, Taiwan; corallab@ntu.edu.tw; 8 College of Medicine and PhD Program in Toxicology, College of Pharmacy, Kaohsiung Medical University, Kaohsiung 807, Taiwan; 9 Institute of Natural Products, Kaohsiung Medical University, Kaohsiung 807, Taiwan; 10 Department of Medical Research, China Medical University Hospital, China Medical University, Taichung 404, Taiwan; 11 Frontier Center for Ocean Science and Technology, National Sun Yat-Sen University, Kaohsiung 804, Taiwan

**Keywords:** soft coral, *Umbellulifera petasites*, steroid, cytotoxic activity, anti-inflammatory activity

## Abstract

Three new steroids, petasitosterones A and B (**1** and **2**) and a spirosteroid petasitosterone C (**3**), along with eight known steroids (**4**–**11**), were isolated from a Formosan marine soft coral *Umbellulifera petasites*. The structures of these compounds were elucidated by extensive spectroscopic analysis and comparison of spectroscopic data with those reported. Compound **3** is a marine steroid with a rarely found A/B spiro[4,5]decane ring system. Compounds **1**–**3** and **5** displayed inhibitory activity against the proliferation of a limited panel of cancer cell lines, whereas **2** and **5** exhibited significant anti-inflammatory activity to inhibit nitric oxide (NO) production. The inhibitory activities for superoxide anion generation and elastase release of compounds **1**–**11** were also examined to evaluate the anti-inflammatory potential, and **2**–**4** were shown to exhibit significant activities.

## 1. Introduction

**Figure 1:**
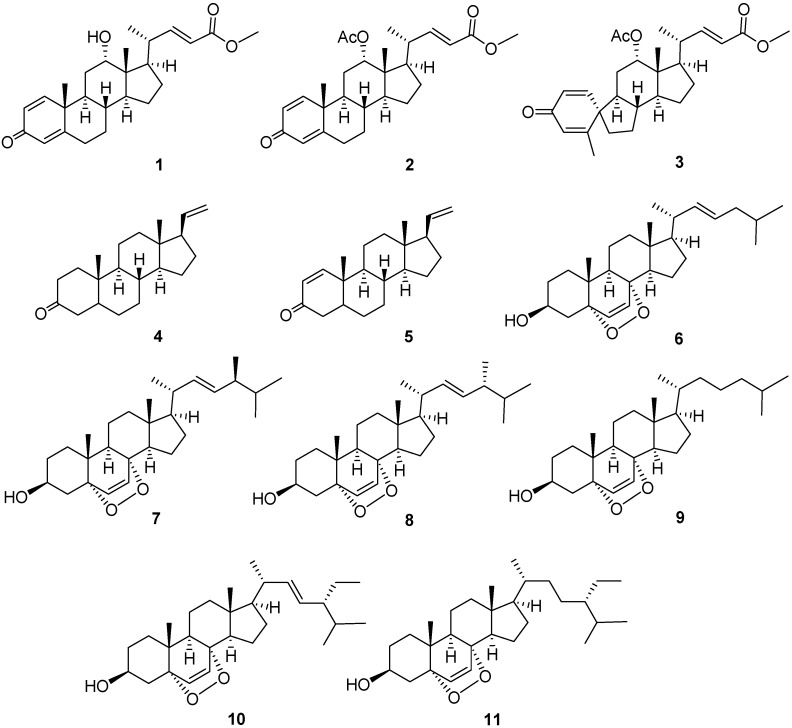
Structures of compounds **1**–**11**.

Marine organisms, including octocorals (Coelenterata: Anthozoa), have been shown to be a rich source of a variety of polyoxygenated steroids [1,2,3,4,5,6,7,8,9,10,11,12,13,14]. Some of these metabolites possess important bioactivities, such as cytotoxic [1,3,5,6,9,11,12,13,14], anti-inflammatory [3,6,8,11,13,14], antiviral [3,4], and antibacterial activities [3,7,10]. In order to discover medicinally useful natural compounds, we investigated the chemical constituents of a soft coral *Umbellulifera petasites*, which was chemically examined for the first time. The study led to the isolation of two new steroids, petasiterones A and B (**1** and **2**), and a novel spirosteroid, petasitosterone C (**3**), along with eight known steroids, 5α-pregna-20-en-3-one (**4**) [15], 5α-pregna-1,20-dien-3-one (**5**) [15], 5α,8α-epidioxycholesta-6,22-dien-3β-ol (**6**) [16], 25α,8α-epidioxy-24(*S*)-methylcholesta-6,22-dien-3β-ol (**7**) [16], 5α,8α-epidioxy-24(*R*)-methylcholesta-6,22-dien-3β-ol (**8**) [16], 5α,8α-epidioxycholest-6-en-3β-ol (**9**) [16], 5α,8α-epidioxy-24α-ethylcholesta-6,22-dien-3β-ol (**10**) [16], and 5α,8α-epidioxy-24α-ethylcholesta-6-en-3β-ol (**11**) [16] (Figure 1). The structures of the new metabolites were determined on the basis of extensive spectroscopic analysis (Supplementary Materials, Figures S1–S9), including HRESIMS and 1D and 2D NMR (COSY, HMQC, HMBC, and NOESY) spectroscopy. With the aim of discovering the bioactivities of the isolated natural products, the anti-inflammatory activity including nitric oxide (NO) inhibition activity of compounds **1**–**11** was evaluated by assay of LPS-stimulated NO production in activated RAW264.7 cells. The ability of suppressing superoxide anion generation and elastase release in *N*-formyl-methionyl-leucyl-phenylalanine/cytochalasin B (fMLP/CB)-induced human neutrophils were also studied. Furthermore, the cytotoxicities of compounds **1**–**11** against the cancer cell lines human erythroleukemia (K-562), lymphoid T carcinoma (MOLT-4), and human colorectal adenocarcinoma (DLD-1) were assayed. We report herein the isolation, structure elucidation, and biological activities of these marine natural products.

## 2. Results and Discussion

**Figure 2:**
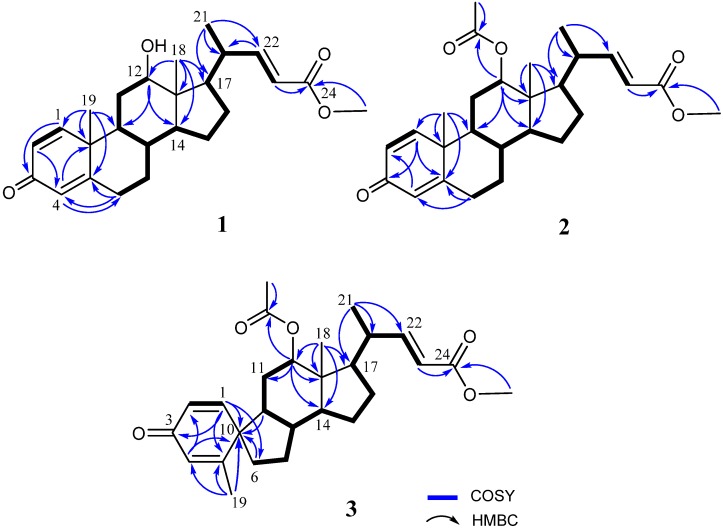
Selected COSY and HMBC correlations of **1**–**3**.

The frozen bodies of *Umbellulifera petasites* were sliced and extracted with ethyl acetate (EtOAc). The EtOAc extracts were evaporated and the residue was repeatedly chromatographed over silica gel and RP-HPLC to afford three new steroids, along with eight known steroids (**4**–**11**). Petasitosterone A (**1**), obtained as an amorphous solid, was found to possess a molecular formula C_25_H_34_O_4_ as established by HRESIMS (*m*/*z* 421.2350, [M + Na]^+^), appropriated for nine degrees of unsaturation. The IR spectrum revealed the presence of hydroxy (3445 cm^−1^), and carbonyl (1715 and 1663 cm^−1^) groups. The ^1^H NMR spectral data (Table 1) of **1** showed the presence of five olefinic methine protons (δ_H_ 6.98, dd, *J* = 15.6, 10.0 Hz; 6.97, d, *J* = 10.4 Hz; 6.21, dd, *J* = 10.4, 2.0 Hz; 6.06, s; 5.83, d, *J* = 15.6 Hz), and one oxymethine proton (δ_H_ 3.74, br s). The ^13^C NMR data (Table 1) and DEPT spectra indicated the presence of 25 carbons, including four methyl groups (containing a methoxy carbon), five methylenes, 11 methines, and five quaternary carbons (including two carbonyl groups). The carbon resonances at δ_C_ 186.4 (C), 155.4 (CH), 127.7 (CH), 123.9 (CH), and 169.0 (C) as well as the proton resonances at δ_H_ 6.97 (1H, d, *J* = 10.4 Hz), 6.21 (1H, dd, *J* = 10.4, 2.0 Hz), and 6.06 (1H, s) were characteristic signals of steroids with a 1,4-dien-3-one moiety in ring A [9]. Careful analysis of the COSY and HMBC spectra (Figure 2) allowed us to determine the molecular skeleton of **1**. H-12 (δ_H_ 3.74, br s) showed HMBC correlations to C-9 and C-14, and H_3_-18 (δ_H_ 0.72, s) exhibited HMBC correlations to C-12, C-13, C-14, and C-17; revealing the position of a hydroxyl at C-12. As C-24 resonated at δ_C_ 167.4, and protons of the methoxyl (δ_H_ 3.74) gave HMBC correlation to this carbonyl carbon, thus the position of the methoxy group at C-24 carbonyl carbon was confirmed. On the basis of the molecular framework, the gross structure of **1** was established (Figure 2).

**Figure 3:**
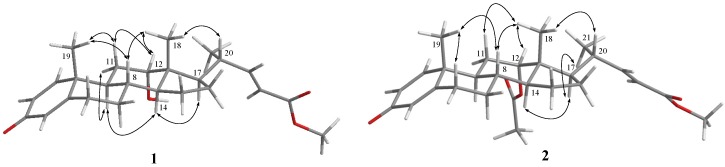
Selected NOE correlations for **1** and **2**.

The relative configuration of **1** was established by the NOE correlations observed in a NOESY experiment. H-8 was found to show NOE correlations with both H_3_-18 and H_3_-19, and H_3_-18 exhibited correlations with one of the methylene protons at C-11 (δ_H_ 1.87, m), H-12, and H-20; therefore, due to the β-orientation of H_3_-18, all of H-8, H-12, H_3_-19, and H-20 should also be positioned on the β-face. Furthermore, NOE responses between H-11α (δ_H_ 1.75, m) and H-9, H-9 and H-14, and H-14 and H-17, were observed on the α-orientation of H-9, H-14 and H-17 (Figure 3).

Metabolite **2** was isolated as an amorphous solid and was found to possess a molecular formula C_27_H_36_O_5_, as established by the HRESIMS *m*/*z* 463.2458 [M + H]^+^ and NMR data (Table 1). The IR absorption bands at ν_max_ 1731 and 1665 cm^−1^ also revealed the presence of carbonyl groups. Comparison of the NMR spectral data of **2** with those of the known metabolite **1** (Table 1) suggested that **2** is the 12-*O*-acetyl derivative of **1**. This was further supported by the downfield shifts observed for H-12 (δ_H_ 4.75, br s) and C-12 (δ_C_ 74.2) relative to those of **1**. The planar structure of **2**, including the positions of acetoxy group, carboxylate, and the olefinic double bond of this metabolite, could be further deduced from the detailed analyses of the COSY, HMQC, and HMBC spectral correlations (Figure 2). Finally, the relative stereochemistry of **2** was established by the analysis of the NOE correlations in NOESY spectrum of **2**, as illustrated in Figure 3.

**Figure 4:**
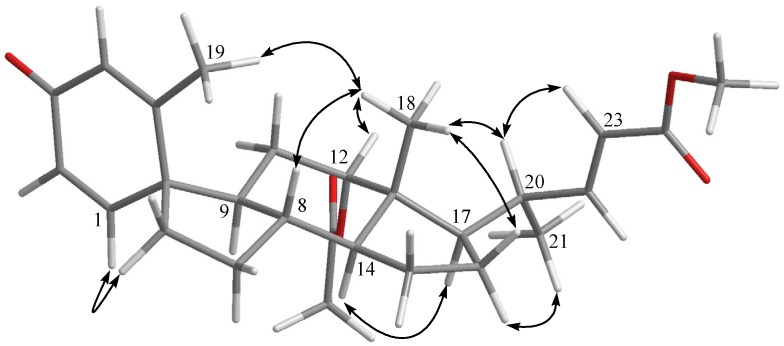
Selected NOE correlations for **3**.

The molecular formula of petasitosterone C (**3**) was found to be C_27_H_36_O_5_ as deduced from HRESIMS and ^13^C NMR data, appropriate for 10 degrees of unsaturation. The IR spectrum of **3** again showed the presence of carbonyl (ν_max_ 1737 and 1661 cm^−1^) groups. The ^13^C NMR and DEPT spectra showed signals of five methyls (including a methoxy carbon), five methylenes, 11 methines, and six quaternary carbons (including two ester carbonyls and one keto-carbonyl). The ^1^H NMR spectrum of **3** exhibited two doublet methyl signals at δ_H_ 1.90 (*J* = 1.2 Hz) and 0.99 (*J* = 6.4 Hz), three singlet methyl signals at δ_H_ 3.71, 2.04, and 0.72, an oxygenated methine group at δ_H_ 4.69, and five olefinic protons at δ_H_ 6.82, 6.61, 6.18, 6.14, and 5.76, respectively. The carbon skeleton of **3** was determined by 2D NMR experiments, in particular the analysis of COSY, HMQC, and HMBC corrections (Figure 2). The COSY correlations from H-1 to H-2 and the HMBC correlations from H-1 to C-3, C-5, C-6, and C-10; H-4 to C-2 and C-10; and H_3_-19 to C-4, C-5, and C-10, suggested a cross-conjugated dienone moiety in **3**. This was further supported by signals of protons at δ_H_ 6.82 (1H, d, *J* = 10.0 Hz), 6.18 (1H, dd, *J* = 10.0, 1.6 Hz), 6.14 (1H, s), and 1.90 (3H, d, *J* = 1.2 Hz). The aforementioned information, along with the HMBC correlations from H-1 to C-3 and C-6, and H-6 to C-9 and C-10, suggested a spiro[4,5]decane ring with a 1,4-diene-3-one partial structure in the A ring of compound **3** [17]. From all of the ^1^H and ^13^C NMR data and other COSY and HMBC correlations, it was found that the rest part of the structure (rings C and D, and side chain) is the same as that of **1**. The configuration of **3** was determined by the correlations observed in a NOESY experiment (Figure 4). The NOE correlations between H-1 and one proton of H_2_-6 (δ_H_ 1.77), and H_3_-19 and H_3_-18, established the β-orientation of C-5, and the α-orientation of C-1. In addition, H_3_-18 was found to show NOE responses with H-12 and H-20, revealing the α-orientation of H_3_-21 the acetoxy group. Steroid **3** is the third natural product possessing a spiro[4,5]decane unit transformed from A and B rings [7,17] and was found to be a compound with a new carbon skeleton after considering the entire molecular framework.

The biosynthesis of **3** might come from the initial protonation of **2** at the carbonyl oxygen of the α,β-unsaturated ketone, followed by the 1,2-shift of the methyl substituent from C-10 to carbonium carbon C-5 and the subsequent 1,2-shift of C-6 residue to C-5, as suggested previously [17].

To find the future biomedical potential for the above steroids, the cytotoxicity of compounds **1**–**11** against the proliferation of a limited panel of cancer cell lines, including human erythroleukemia (K-562), lymphoid T carcinoma (MOLT-4), and human colorectal adenocarcinoma (DLD-1), was evaluated. The results showed compound **5** exhibited cytotoxicity toward K-562, MOLT-4, and DLD-1 cancer cell lines with IC_50_ values of 13.5 ± 3.1, 5.9 ± 1.9, and 9.7 ± 3.2 μg/mL, respectively, while **2** was found to show cytoxicity toward MOLT-4 and DLD-1 with IC_50_ values of 12.1 ± 4.5 and 5.8 ± 1.7 μg/mL. Also, **1** and **3** showed cytotoxicity toward the DLD-1 cell line, as show in Table 2.

Compounds **1**–**11** were also evaluated for anti-inflammatory activity by suppressing superoxide anion generation and elastase release by human neutrophils in response to fMLP/CB stimulation. The results revealed that compounds **2** and **3** showed moderate activities toward superoxide anion generation with IC_50_ values of 4.43 ± 0.23 and 2.76 ± 0.92 μM, respectively. Compound **4** did not exhibit inhibition activity toward superoxide anion generation (IC_50_ > 10 μM), but significantly inhibited the fMLP/CB-induced elastase release with IC_50_ value of 6.80 ± 0.18 μM (Table 3).

**Figure 5:**
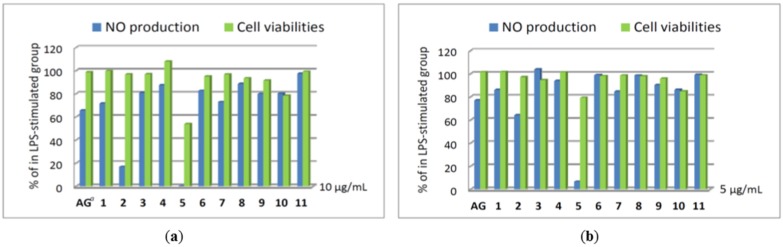
Nitric oxide (NO) production and cell viabilities of compounds **1**–**11** in LPS-stimulated RAW264.7 cells (**a**) at 10 μg/mL (**b**) at 5 μg/mL. ^a^ AG: aminoguanidine used as a positive control.

In addition, the nitric oxide (NO) inhibitory activities of compounds **1**–**11** were further evaluated by assay of LPS-stimulated NO production in activated RAW264.7 cells, as shown in Figure 5. The results indicated that compound **5** could effectively reduce the level of NO to 6.6% at a concentration of 5 μg/mL, with 79.3% retention of cell viability. Moreover, compounds **2** and **5** at the concentration of 10 μg/mL exhibited good inhibitory activity compared to the positive control aminoguanidine (AG), with the levels of NO reduced significantly to 16.9% and 0.3%, respectively, while giving 96.8% and 53.9% retention of cell viability. Thus, compounds **2** and **5** are promising metabolites that might become lead compounds in future anti-inflammatory drug development.

## 3. Experimental Section

### 3.1. General Experimental Procedures

Optical rotations were measured on a JASCO P-1020 digital polarimeter (JASCO Corporation, Tokyo, Japan). IR spectra were recorded on a JASCO J-815 spectrophotometer (JASCO Corporation, Tokyo, Japan). Ultraviolet spectra were recorded on a JASCO V-650 spectrophotometer (JASCO Corporation, Tokyo, Japan). The ^1^H NMR and ^13^C NMR spectra were recorded on Varian 400MR NMR (400 MHz for ^1^H and 100 MHz for ^13^C) instruments (Varian Inc., Palo Alto, CA, USA). The chemical shifts were referenced to the solvent residue of CDCl_3_ (δ_H_ 7.265 ppm and δ_C_ 77.0 ppm). The ESIMS and HRESIMS were acquired via a Bruker APEX II mass spectrometer with an ESI ionization source (Bruker, Bremen, Germany). Silica gel 60 (40–63 μm, Merck, Darmstadt, Germany), and C18 gel (LiChroprep RP-18, 40–63 μm, Merck, Darmstadt, Germany) were used for column chromatography. TLC analysis was performed on precoated silica gel plates (Kieselgel 60 F_254_, 0.25 mm, Merck, Darmstadt, Germany). High-performance liquid chromatography (HPLC) was performed using a Shimadzu LC-10AT*_VP_* series pump equipped with a UV detector and a semipreparative RP-18 column (5 μm, 250 mm × 10 mm, Hibar Purospher RP-18e, Merck, Darmstadt, Germany).

### 3.2. Animal Material

The soft coral *Umbellulifera petasites* was collected by hand using scuba on reefs at depths of 10–15 m, along the coast of Kaohsiung, located at Southern Taiwan in October 2008. The material was frozen at −20 °C until extraction in the laboratory. Species identification of this coral was performed by C.-F. Dai (National Taiwan University, Taipei, Taiwan).

### 3.3. Extraction and Isolation

The frozen bodies of *U. petasites* (1.2 kg, wet wt) were sliced and exhaustively extracted with EtOAc (3 × 2 L). The EtOAc extract (12.6 g) was chromatographed over silica gel by column chromatography and eluting with EtOAc in *n*-hexane (0%–100%, stepwise) then with MeOH in EtOAc (5%–50%, stepwise) to yield 24 fractions. Fraction 11, eluting with *n*-hexane–EtOAc (15:1), was further purified over silica gel using *n*-hexane–EtOAc (13:1) to yield compounds **4** (20.0 mg) and **5** (53.0 mg). Fraction 15, eluting with *n*-hexane–EtOAc (10:1), was further purified by reversed-phase HPLC using MeOH–H_2_O (15:1) to afford **6** (0.9 mg), **7** (2.5 mg), **8** (1.1 mg), **9** (0.8 mg), **10** (1.5 mg), and **11** (0.9 mg). Fraction 16, eluting with *n*-hexane–EtOAc (9:1), was further purified by reversed-phase HPLC using acetonitrile–H_2_O (5:1) to afford **1** (1.5 mg), **2** (13.0 mg), and **3** (1.2 mg).

Petasitosterone A (**1**): amorphous solid; ${\left[\alpha \right]}_{D}^{28}$ +186 (*c* 0.375, CHCl_3_); UV (MeOH) λ_max_ (log ε) 241 (4.4) and 220 (4.4); IR (neat) ν_max_ 3445, 2948, 2871, 1715, 1653, 1437, 1341 and 1246 cm^−1^; ^13^C and ^1^H NMR data, Table 1; ESIMS *m*/*z* 421 [M + Na]^+^; HRESIMS *m*/*z* 421.23500 [M + Na]^+^ (calcd. for C_25_H_34_O_4_Na, 421.23493).

Petasitosterone B (**2**): amorphous solid; ${\left[\alpha \right]}_{D}^{28}$ +139 (*c* 3.25, CHCl_3_); UV (MeOH) λ_max_ (log ε) 242 (3.9) and 217 (3.9); IR (neat) ν_max_ 2967, 2873, 1731, 1665, 1625, 1435, 1270 and 1241 cm^–1^; ^13^C and ^1^H NMR data, Table 1; ESIMS *m*/*z* 463 [M + Na]^+^; HRESIMS *m*/*z* 463.24579 [M + Na]^+^ (calcd. for C_27_H_36_O_5_Na, 463.24550). 

Petasitosterone C (**3**): amorphous solid; ${\left[\alpha \right]}_{D}^{28}$ −8 (*c* 0.300, CHCl_3_); UV (MeOH) λ_max_ (log ε) 243 (4.1) and 206 (4.3); IR (neat) ν_max_ 2952, 2871, 1737, 1661 and 1241 cm^–1^; ^13^C and ^1^H NMR data, Table 1; ESIMS *m*/*z* 463 [M + Na]^+^; HRESIMS *m*/*z* 463.24524 [M + Na]^+^ (calcd. for C_27_H_36_O_5_Na, 463.24550).

5α-Pregna-20-en-3-one (**4**): amorphous solid; ${\left[\alpha \right]}_{D}^{25}$ +15 (*c* 0.20, CHCl_3_); lit. ${\left[\alpha \right]}_{D}^{25}$ +12.5 (*c* 0.20, CHCl_3_); MS, ^1^H and ^13^C NMR data were found to be in full agreement with those reported previously [15].

5α-Pregna-1,20-dien-3-one (**5**)*:* amorphous solid; ${\left[\alpha \right]}_{D}^{25}$ +35 (*c* 0.50, CHCl_3_); lit. ${\left[\alpha \right]}_{D}^{25}$ +35.4 (*c* 0.50, CHCl_3_); MS, ^1^H and ^13^C NMR data were found to be in full agreement with those reported previously [15].

5α,8α-Epidioxycholesta-6,22-dien-3β-ol (**6**): amorphous solid; ${\left[\alpha \right]}_{D}^{25}$ +95 (*c* 0.50, CHCl_3_); MS, ^1^H and ^13^C NMR data were found to be in full agreement with those reported previously [16].

25α,8α-Epidioxy-24(*S*)-methylcholesta-6,22-dien-3β-ol (**7**): amorphous solid; ${\left[\alpha \right]}_{D}^{25}$ +13 (*c* 0.20, CHCl_3_); MS, ^1^H and ^13^C NMR data were found to be in full agreement with those reported previously [16].

5α,8α-Epidioxy-24(*R*)-methylcholesta-6,22-dien-3β-ol (**8**): amorphous solid; ${\left[\alpha \right]}_{D}^{25}$ −8 (*c* 0.16, CHCl_3_); MS, ^1^H and ^13^C NMR data were found to be in full agreement with those reported previously [16].

5α,8α-Epidioxycholest-6-en-3β-ol (**9**): amorphous solid; ${\left[\alpha \right]}_{D}^{25}$ −30 (*c* 0.50, CHCl_3_); MS, ^1^H and ^13^C NMR data were found to be in full agreement with those reported previously [16].

5α,8α-Epidioxy-24α-ethylcholesta-6,22-dien-3β-ol (**10**): amorphous solid; ${\left[\alpha \right]}_{D}^{25}$ −6 (*c* 0.50, CHCl_3_); MS, ^1^H and ^13^C NMR data were found to be in full agreement with those reported previously [16].

5α,8α-Epidioxy-24α-ethylcholesta-6-en-3β-ol (**11**): amorphous solid; ${\left[\alpha \right]}_{D}^{25}$ −27 (*c* 0.50, CHCl_3_); MS, ^1^H and ^13^C NMR data were found to be in full agreement with those reported previously [16].

### 3.4. Cytotoxicity Assay

The Alamar Blue assays were performed as previous reported [18,19]. After the cell lines (K-562, MOLT-4, and DLD-1) were cultured for 15 h according to the published procedure [20], the tested compounds in DMSO solutions were added and cultured for 72 h. The attached cells were incubated with Alamar Blue (10 μL/well, 4 h) and the absorbance was measured at wavelength of 595 nm using a microplate reader.

### 3.5. Human Neutrophil Superoxide Anion Generation and Elastase Release

The human neutrophils were isolated using a standard method of dextran sedimentation and Ficoll centrifugation [21,22]. As in previously described procedures, the assay of superoxide anion generation was conducted according to the SOD-inhibitable reduction of ferricytochrome C. The elastase release experiment was performed using MeO–Suc–Ala–Ala–Pro–Val–*p*-nitroanilide as the enzyme substrate [23]. Idelalisib, a selective inhibitor of phosphatidylinositol-3-kinase, was used as a positive control for inhibition of superoxide anion generation and elastase release with IC_50_ 0.07 ± 0.01 and 0.3 ± 0.1 μM [24].

### 3.6. Nitric Oxide Inhibitory Activity

The nitrite concentration in the culture medium was measured as an indicator of NO production according to the Griess reaction [25]. Briefly, 80 μL of cell culture supernatant was reacted with 100 μL of Griess reagent (1:1 mixture of 0.1% *N*-(1-naphthyl)ethylenediamine dihydrochloride in water and 1% sulfanilamide in 5% phosphoric acid) in a 96-well plate and incubated at room temperature for 10 min. The absorbance at 550 nm was recorded using the ELISA reader [26,27]. Fresh medium was used as the blank. The results are expressed as the percentage of inhibition calculated relative to the cells treated with vehicle and LPS.

### 3.7. Statistical Analysis

Results are expressed as the mean ± SEM, and comparisons were made using Student’s *t*-test. A probability value of 0.05 or less was considered significant. The software SigmaPlot was used for the statistical analysis.

## 4. Conclusions

*Umbellulifera petasites* (Thomson and Dean, 1931) [28], which is here investigated for the first time, afforded three new steroids petasitosterones A–C (**1**–**3**), along with eight known steroids **4**–**11**. It is worthwhile to mention that compound **3** represents a novel steroid with an A/B spiro[4,5]decane ring system. Our present study shows that compounds **1**–**3** and **5** exhibited significant cytotoxicity toward a limited panel of cancer cell lines. Moreover, compounds **2**–**5** are promising compounds, which have displayed potent anti-inflammatory activity in different assays.

## Figures and Tables

**Table 1:** ^1^H and ^13^C NMR spectroscopic data of **1**–**3**.

	1		2		3	
Position	δ_C_ (mult.) ^a^	δ_H_ (*J* in Hz) ^b^	δ_C_ (mult.) ^a^	δ_H_ (*J* in Hz) ^b^	δ_C_ (mult.) ^a^	δ_H_ (*J* in Hz) ^b^
1	155.4, CH ^c^	6.97 d (10.4)	155.0, CH	6.88 d (10.0)	153.3, CH	6.82 d (10.0)
2	127.7, CH	6.21 dd (10.4, 2.0)	127.8, CH	6.19 dd (10.0, 2.0)	127.1, CH	6.18 dd (10.0, 1.6)
3	186.4, C		186.3, C		186.0, C	
4	123.9, CH	6.06 s	124.0, CH	6.06 s	129.1, CH	6.14 s
5	169.0, C		168.5, C		161.8, C	
6	32.8, CH_2_	2.45 td (13.6, 4.0)	32.7, CH_2_	2.45 td (13.6, 4.0)	34.9, CH_2_	1.99 m
		2.38 m		2.38 m		1.77 m
7	33.3, CH_2_	1.95 m	33.2, CH_2_	1.97 m	30.1, CH_2_	2.01 m
		1.10 m		1.07 m		1.40 m
8	35.6, CH	1.63 m	35.4, CH	1.64 m	42.0, CH	1.81 m
9	46.4, CH	1.47 m	46.6, CH	1.23 m	49.8, CH	1.80 m
10	43.0, C		42.9, C		51.8, C	
11	30.6, CH_2_	1.75 m	26.8, CH_2_	2.01 m	26.7, CH_2_	1.45 m
		1.87 m		1.77 m		1.35 m
12	70.9, CH	3.79 br s	74.2, CH	4.75 br s	74.0, CH	4.69 br s
13	45.9, C		45.1, C		46.3, C	
14	47.0, CH	1.91 m	48.1, CH	1.54 m	49.5, CH	1.70 m
15	23.6, CH_2_	1.67 m	23.2, CH_2_	1.68 m	23.6, CH_2_	1.72 m
		1.17 m		1.19 m		1.29 m
16	26.7, CH_2_	1.91 m	26.2, CH_2_	1.90 m	26.4, CH_2_	1.94 m
		1.32 m		1.36 m		1.42 m
17	46.7, CH	1.53 m	47.5, CH	1.81 m	47.3, CH	1.79 m
18	12.7, CH_3_	0.72 s	12.8, CH_3_	0.79 s	12.8, CH_3_	0.72 s
19	18.5, CH_3_	1.20 s	18.4, CH_3_	1.18 s	19.2, CH_3_	1.90 d (1.2)
20	39.8, CH	2.21 m	38.6, CH	2.24 m	38.6, CH	2.24 m
21	20.0, CH_3_	1.00 d (6.8)	19.7, CH_3_	0.98 d (6.4)	19.9, CH_3_	0.99 d (6.4)
22	155.0, CH	6.98 dd (15.6, 10.0)	153.3, CH	6.61 dd (15.6, 10.0)	153.2, CH	6.61 dd (15.6, 9.6)
23	119.0, CH	5.83 d (15.6)	119.6, CH	5.77 d (15.6)	119.7, CH	5.76 d (15.6)
24	167.4, C		166.6, C		166.6, C	
OMe	51.6, CH_3_	3.74 s	51.4, CH_3_	3.73 s	51.5, CH_3_	3.71 s
OAc					169.3, C	
					21.0, CH_3_	2.04 s

^a^ Spectrum recorded at 100 MHz in CDCl_3_; ^b^ Spectrum recorded at 400 MHz in CDCl_3_; ^c^ Attached protons were deduced by DEPT experiment.

**Table 2:** Cytotoxicity (IC_50_ μg/mL) of compounds **1**–**3** and **5**.

Compound	Cell Lines IC_50_ (μg/mL)		
	K-562	MOLT-4	DLD-1
**1**	─ ^b^	─	6.4 ± 1.4
**2**	─	12.1 ± 4.5	5.8 ± 1.7
**3**	─	─	15.2 ± 3.5
**5**	13.5 ± 3.1	5.9 ± 1.9	9.7 ± 3.2
Doxorubicin ^a^	0.45 ± 0.08	0.005 ± 0.02	0.2 ± 0.1

^a^ Clinical anticancer drug used as a positive control; ^b^ ─: IC_50_ > 40 μg/mL. Results are presented as mean ± S.E.M. (*n* = 3–5).

**Table 3:** Effect of **1**–**11** on superoxide anion generation and elastase release in fMLP/CB induced human neutrophils.

Compound	Superoxide Anion	Elastase Release
	IC_50_ (μM) ^a^	IC_50_ (μM) ^a^
**1**	>10 ***	>10
**2**	4.43 ± 0.23 ***	>10 ***
**3**	2.76 ± 0.92 ***	>10 *
**4**	>10	6.80 ± 0.18 ***
**5**	>10	>10 ***
**6**	>10	>10
**7**	>10	>10
**8**	>10	>10
**9**	>10	>10
**10**	>10	>10
**11**	>10	>10 ***

^a^ Concentration necessary for 50% inhibition (IC_50_). Results are presented as mean ± S.E.M. (*n* = 3–5). * *p* < 0.05, *** *p* < 0.001 compared with the control.

## Supplementary Materials

HRESIMS, ^1^H, and ^13^C spectra of new compounds **1**–**3** are available online at www.mdpi.com/1660-3397/14/10/180/s1. Figure S1: HRESIMS spectrum of **1**, Figure S2: ^1^H NMR spectrum of **1** in CDCl_3_ at 400 MHz, Figure S3: ^13^C NMR spectrum of **1** in CDCl_3_ at 100 MHz, Figure S4: HRESIMS spectrum of **2**, Figure S5: ^1^H NMR spectrum of **2** in CDCl_3_ at 400 MHz, Figure S6: ^13^C NMR spectrum of **2** in CDCl_3_ at 100 MHz, Figure S7: HRESIMS spectrum of **3**, Figure S8: ^1^H NMR spectrum of **3** in CDCl_3_ at 400 MHz, Figure S9: ^13^C NMR spectrum of **3** in CDCl_3_ at 100 MHz.
