# Supplementary material for: Bioactive Steroids from the Formosan Soft Coral Umbellulifera petasites
Source: Mar Drugs. 2016 Oct 11;14(10):180. doi: 10.3390/md14100180 (PMC5082328; doi:10.3390/md14100180)
Supplement: Supplementary file 1 [file marinedrugs-14-00180-s001.pdf]

Supplementary Materials: Bioactive Steroids from the Formosan Soft Coral *Umbellulifera petasites*

Chiung-Yao Huang, Che-Wei Chang, Yen-Ju Tseng, Jessica Lee, Ping-Jyun Sung, Jui-Hsin Su, Tsong-Long Hwang, Chang-Feng Dai, Hui-Chun Wang and Jyh-Horng Sheu

List of Supplementary material:

No	Content	Page
**Figure S1**	HRESIMS spectrum of **1**.	2
**Figure S2**	^{1}H NMR spectrum of **1** in $CDCl_3$ at 400 MHz.	3
**Figure S3**	^{13}C NMR spectrum of **1** in $CDCl_3$ at 100 MHz.	4
**Figure S4**	HRESIMS spectrum of **2**.	5
**Figure S5**	^{1}H NMR spectrum of **2** in $CDCl_3$ at 400 MHz.	6
**Figure S6**	^{13}C NMR spectrum of **2** in $CDCl_3$ at 100 MHz.	7
**Figure S7**	HRESIMS spectrum of **3**.	8
**Figure S8**	^{1}H NMR spectrum of **3** in $CDCl_3$ at 400 MHz.	9
**Figure S9**	^{13}C NMR spectrum of **3** in $CDCl_3$ at 100 MHz.	10

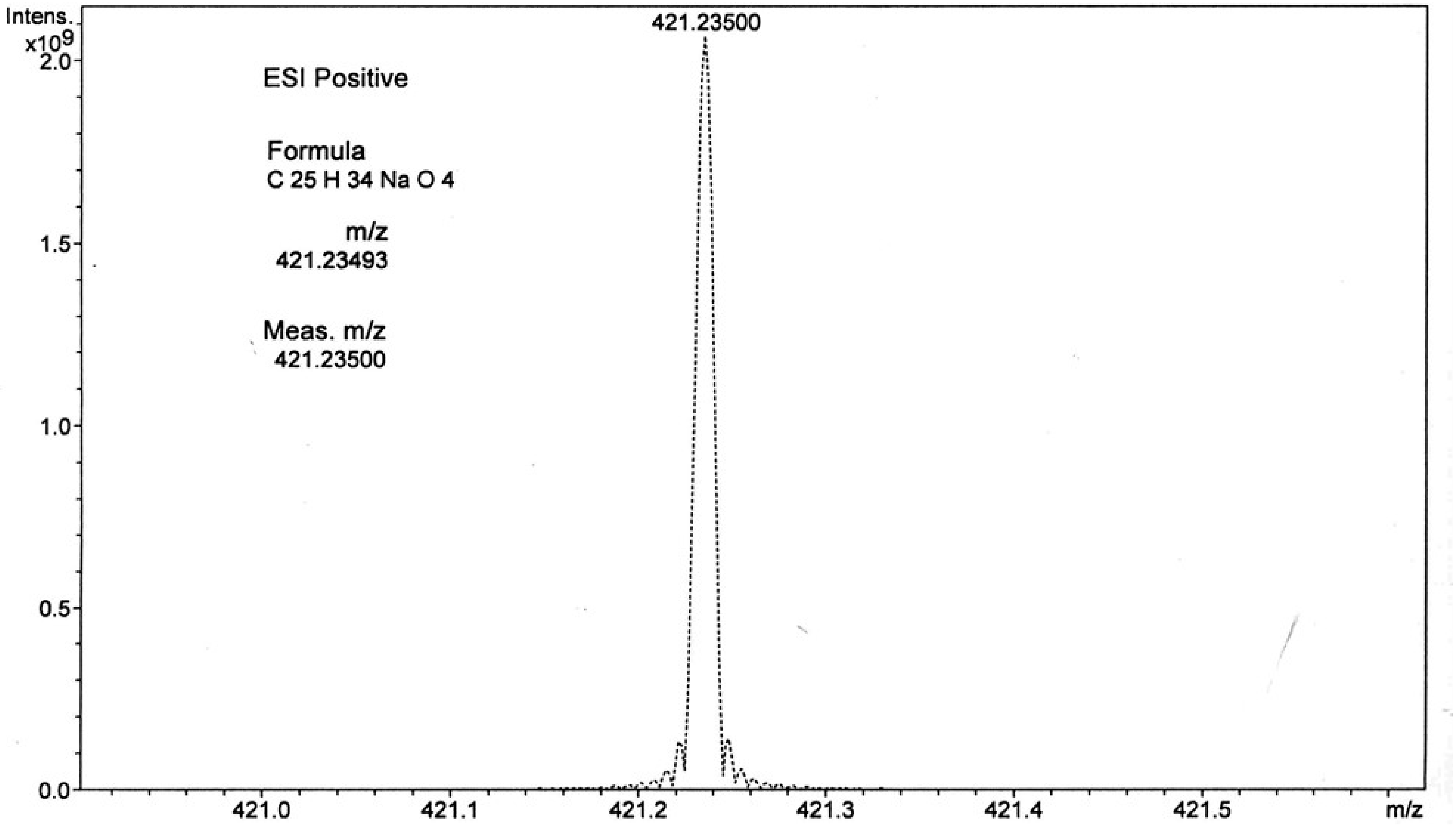

Figure S1. HRESIMS spectrum of **1**.

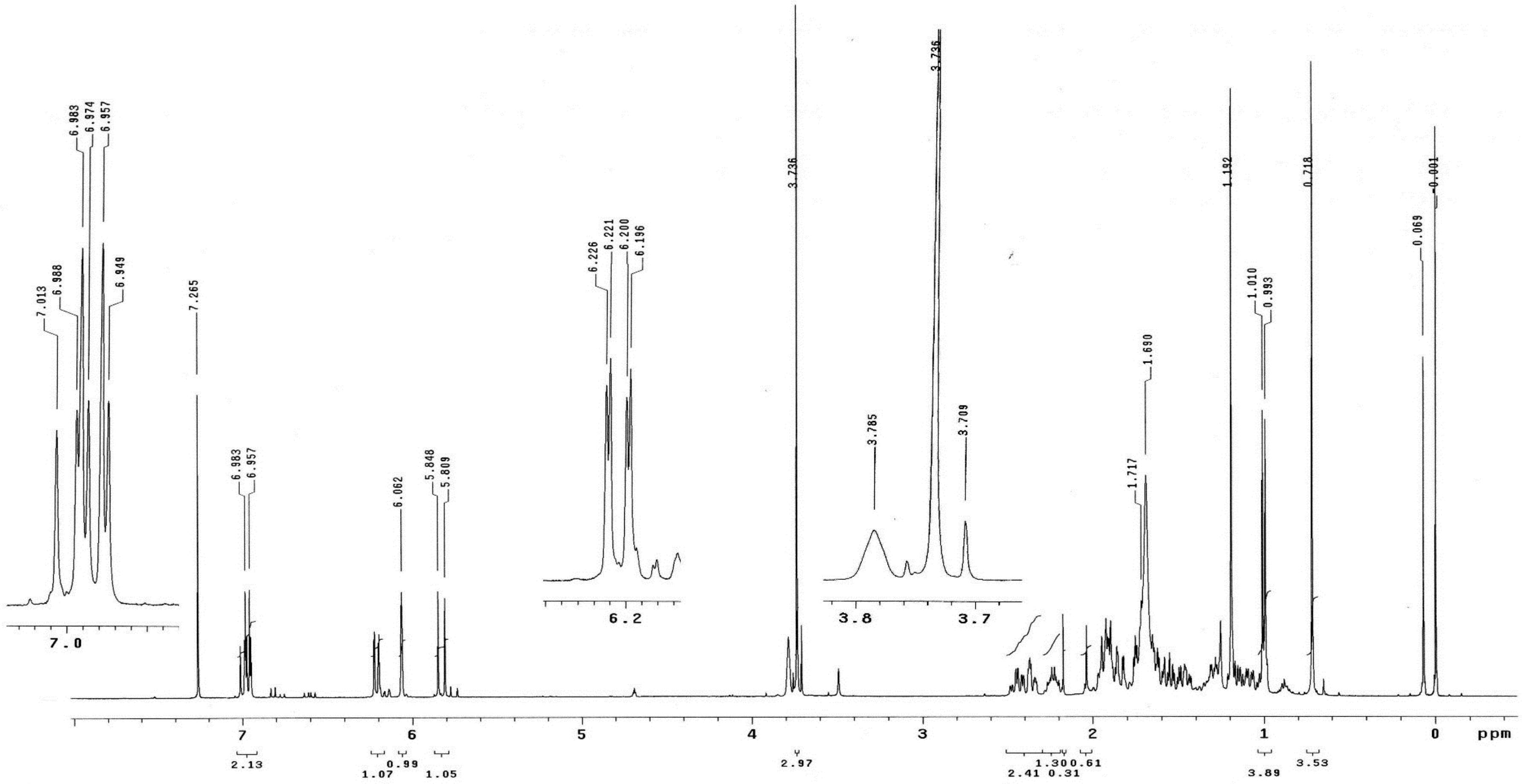

Figure S2. ^{1}H NMR spectrum of **1** in $CDCl_3$ at 400 MHz.

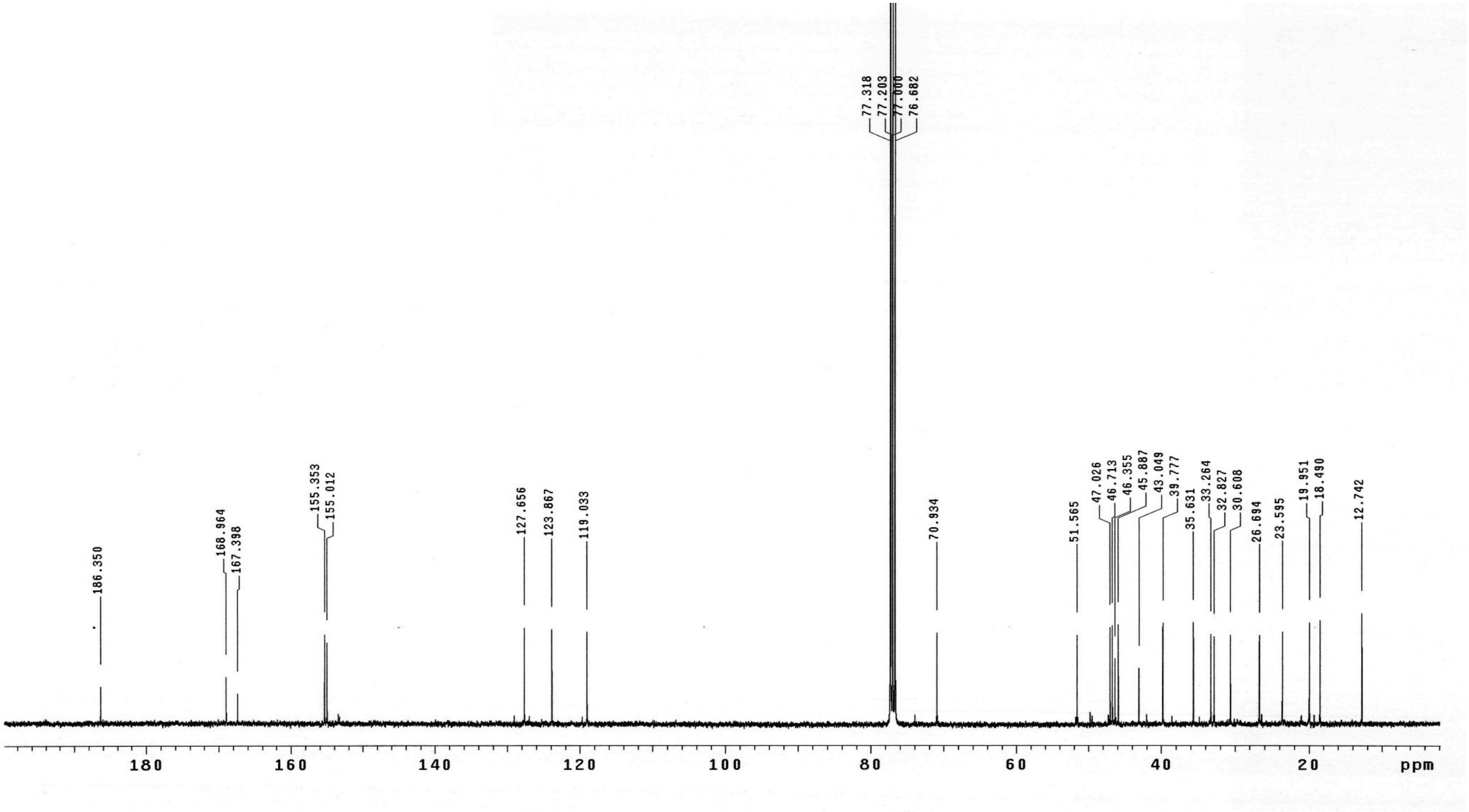

Figure S3. ^{13}C NMR spectrum of **1** in $CDCl_3$ at 100 MHz.

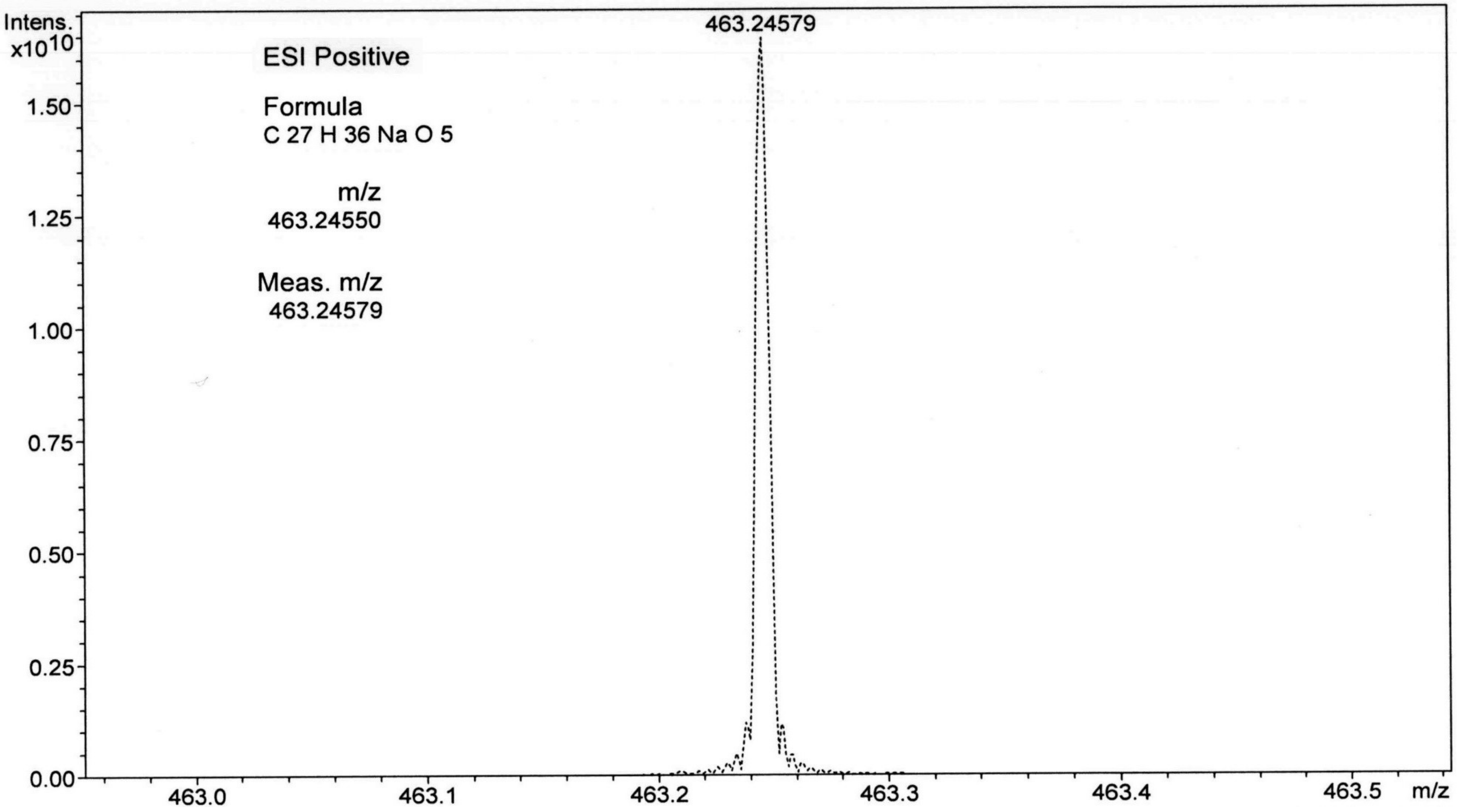

Figure S4. HRESIMS spectrum of **2**.

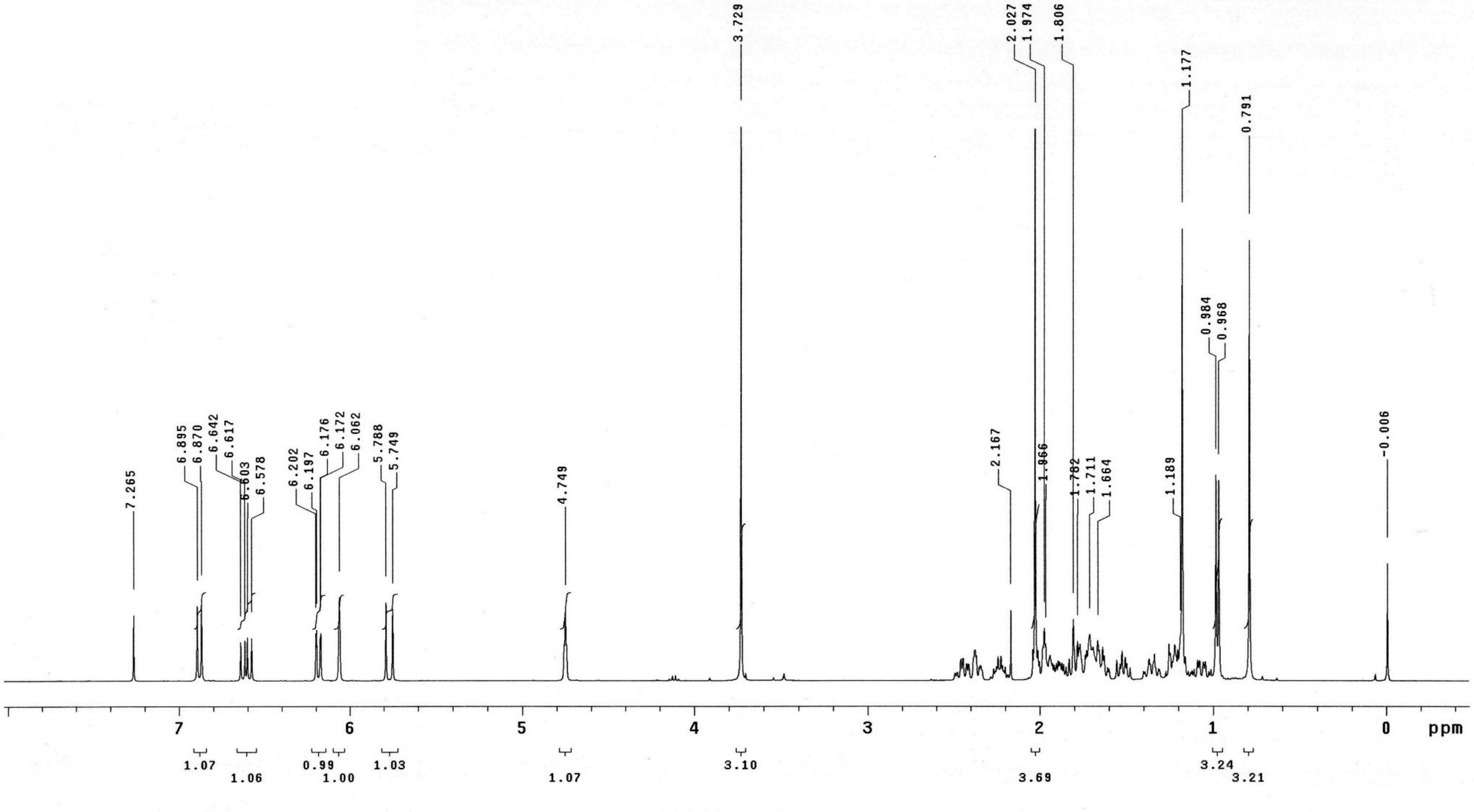

Figure S5. ^{1}H NMR spectrum of **2** in $CDCl_3$ at 400 MHz.

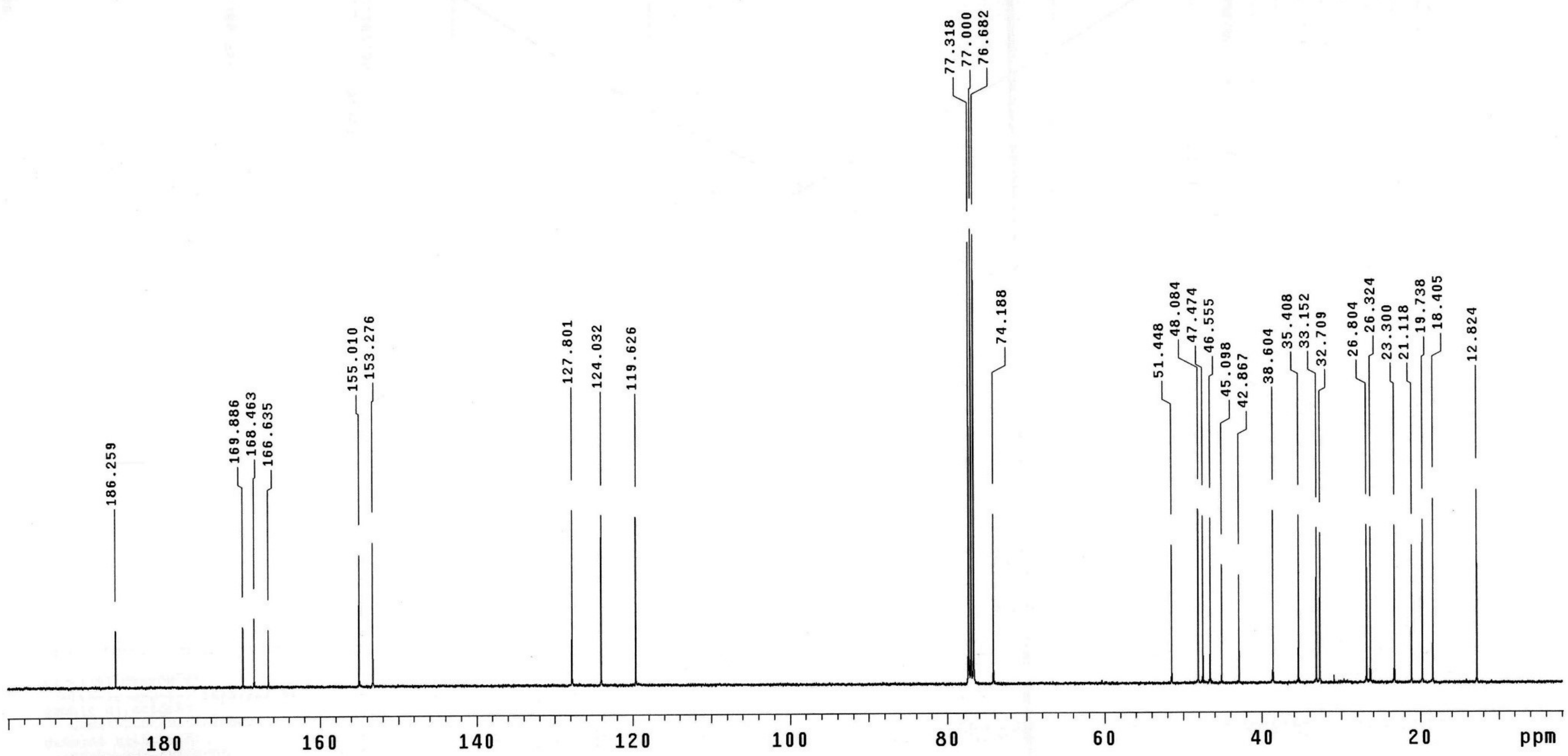

Figure S6. ^{13}C NMR spectrum of **2** in $CDCl_3$ at 100 MHz.

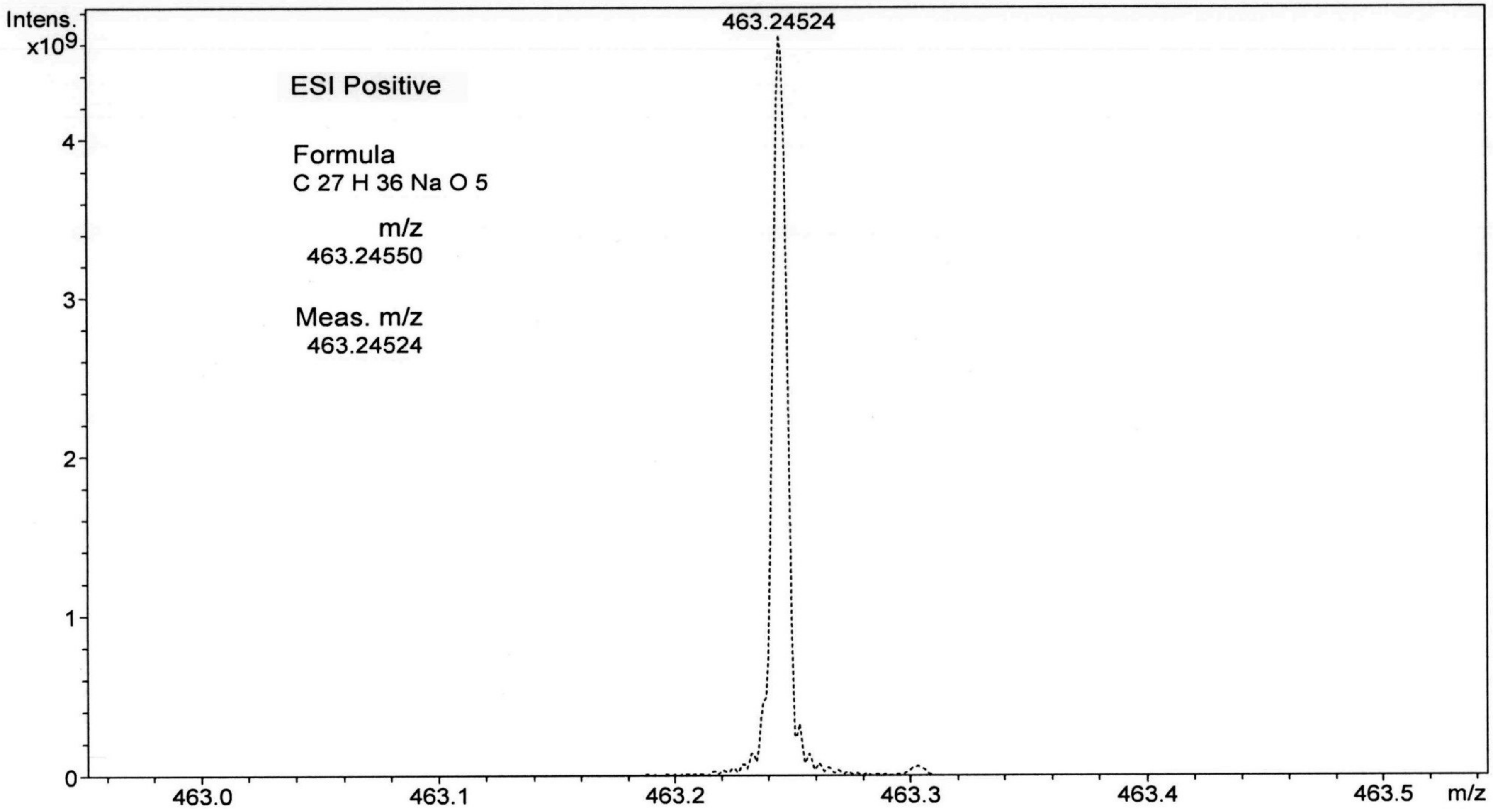

Figure S7. HRESIMS spectrum of **3**.

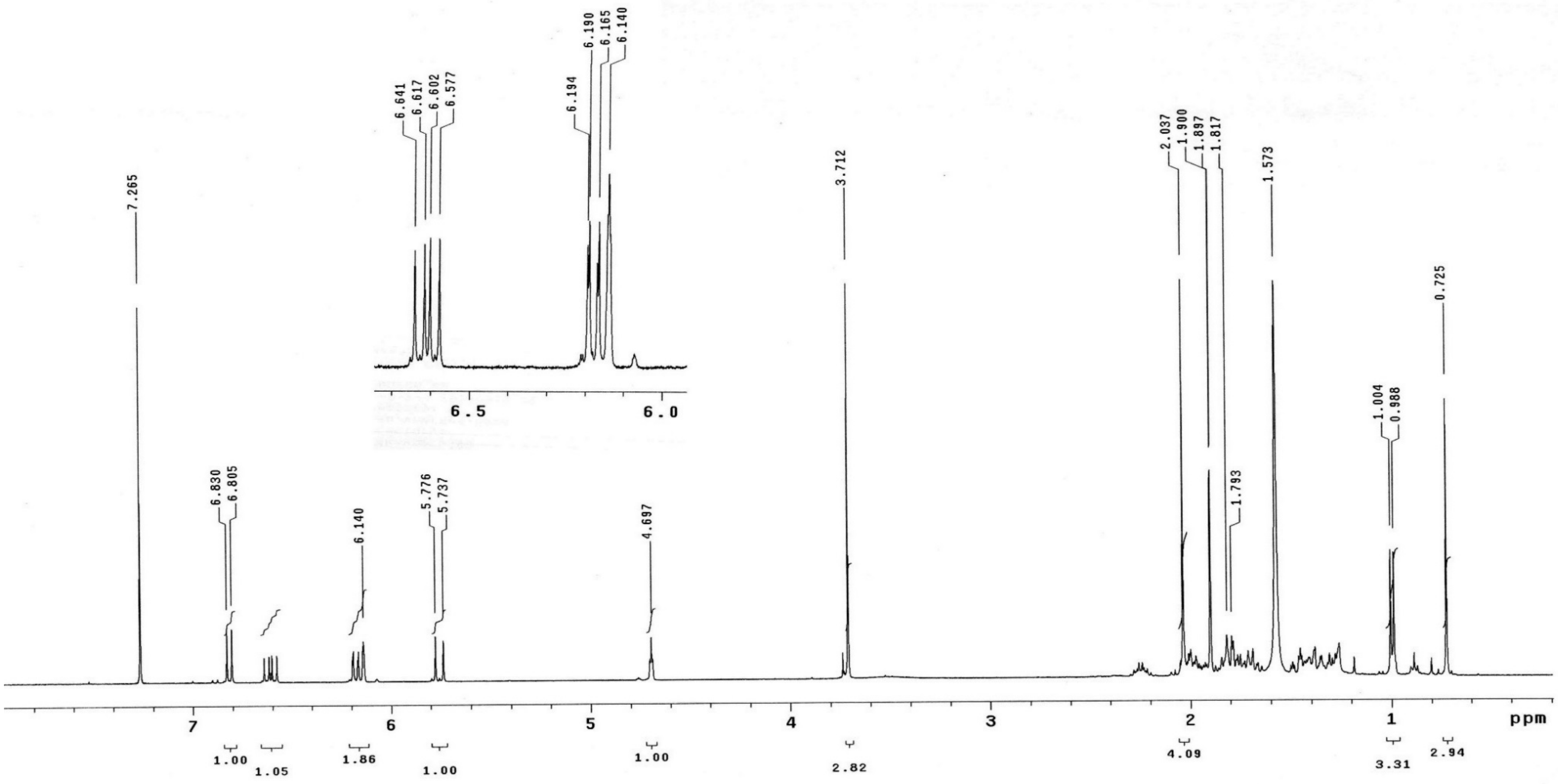

Figure S8. ^{1}H NMR spectrum of **3** in $CDCl_3$ at 400 MHz.

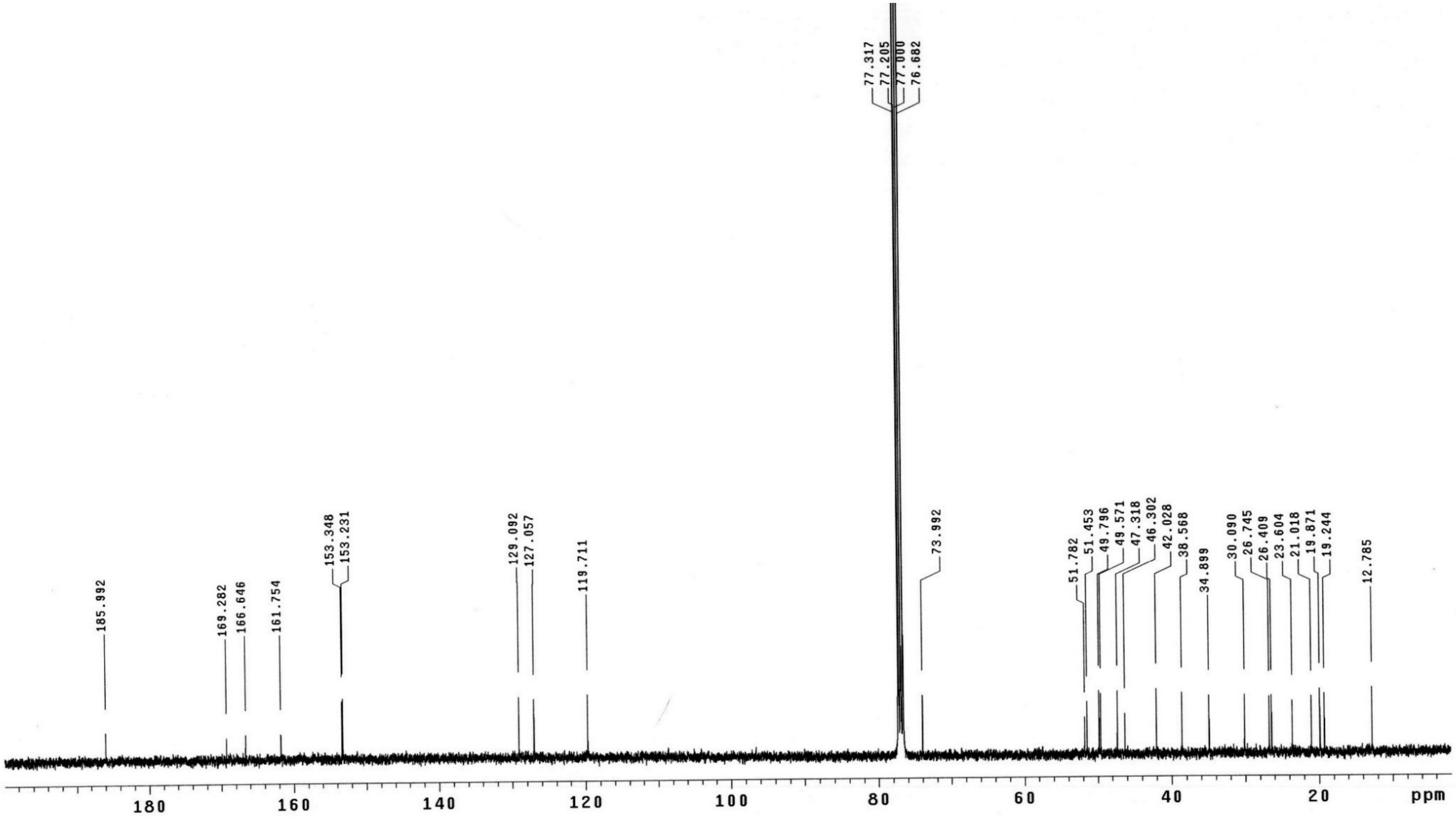

Figure S9. ^{13}C NMR spectrum of **3** in $CDCl_3$ at 100 MHz.
